# Autotaxin, a synthetic enzyme of lysophosphatidic acid (LPA), mediates the induction of nerve-injured neuropathic pain

**DOI:** 10.1186/1744-8069-4-6

**Published:** 2008-02-08

**Authors:** Makoto Inoue, Lin Ma, Junken Aoki, Jerold Chun, Hiroshi Ueda

**Affiliations:** 1 Division of Molecular Pharmacology and Neuroscience, Nagasaki University, Graduate School of Biomedical Sciences, 1-14 Bunkyo-machi, Nagasaki 852-8521, Japan; 2 Laboratory of Molecular and Cellular Biochemistry, Graduate School of Pharmaceutical Sciences, Tohoku University, Sendai, Japan; 3 The Scripps Research Institute, 10550 North Torrey Pines Road, ICND118, La Jolla, CA 92037, USA

## Abstract

Recently, we reported that lysophosphatidic acid (LPA) induces long-lasting mechanical allodynia and thermal hyperalgesia as well as demyelination and upregulation of pain-related proteins through one of its cognate receptors, LPA_1_. In addition, mice lacking the LPA_1 _receptor gene (*lpa*_*1*_^-/- ^mice) lost these nerve injury-induced neuropathic pain behaviors and phenomena. However, since *lpa*_*1*_^-/- ^mice did not exhibit any effects on the basal nociceptive threshold, it is possible that nerve injury-induced neuropathic pain and its machineries are initiated by LPA via defined biosynthetic pathways that involve multiple enzymes. Here, we attempted to clarify the involvement of a single synthetic enzyme of LPA known as autotaxin (ATX) in nerve injury-induced neuropathic pain. Wild-type mice with partial sciatic nerve injury showed robust mechanical allodynia starting from day 3 after the nerve injury and persisting for at least 14 days, along with thermal hyperalgesia. On the other hand, heterozygous mutant mice for the *autotaxin *gene (*atx*^+/-^), which have 50% ATX protein and 50% lysophospholipase D activity compared with wild-type mice, showed approximately 50% recovery of nerve injury-induced neuropathic pain. In addition, hypersensitization of myelinated A$\tilde{\beta }$- or Aδ-fiber function following nerve injury was observed in electrical stimuli-induced paw withdrawal tests using a Neurometer^®^. The hyperalgesia was completely abolished in *lpa*_*1*_^-/- ^mice, and reduced by 50% in *atx*^+/- ^mice. Taken together, these findings suggest that LPA biosynthesis through ATX is the source of LPA for LPA_1 _receptor-mediated neuropathic pain. Therefore, targeted inhibition of ATX-mediated LPA biosynthesis as well as LPA_1 _receptor and its downstream pathways may represent a novel way to prevent nerve injury-induced neuropathic pain.

## Findings

Lysophosphatidic acid (LPA) is a representative lipid mediator that has a variety of biological actions, including roles in cell proliferation, migration and survival via its cognate receptors LPA_1_/EDG2, LPA_2_/EDG4 and LPA_3_/EDG7 [1-4]. Mice lacking LPA_1 _receptor do not develop any signs of neuropathic pain, demyelination or upregulation of pain-related gene/protein expression following nerve injury [5]. Nerve injury-induced neuropathic pain and its underlying machineries are caused by a single intrathecal (i.t.) injection of LPA, and blocked by knockdown of LPA_1 _receptor at the early, but not late, stage. These findings suggest that LPA_1 _receptor activation initiates the machineries of neuropathic pain. Furthermore, since deletion of the LPA_1 _receptor gene did not have any effect on the basal nociceptive threshold, it is evident that nerve injury-induced neuropathic pain and its machineries are initiated by LPA via defined biosynthetic pathways that involve multiple enzymes [6,7]. Therefore, targeted inhibition of LPA biosynthesis as well as LPA_1 _receptor would be a valuable way to prevent nerve injury-induced neuropathic pain. Autotaxin (ATX), which was originally identified as a tumor cell motility factor, is known to have lysophospholipase D (lysoPLD) activity and convert lysophosphatidylcholine (LPC) to LPA [8,9]. Here, we report the involvement of ATX in the development of partial sciatic nerve injury-induced neuropathic pain.

Male heterozygous mutant mice for the *autotaxin *gene (*atx*^+/-^) [10] and mutant mice for the *lpa*_*1 *_gene (*lpa*_*1*_^-/-^) [11], which were backcrossed with C57BL/6J mice at least ten times before use, and their sibling wild-type mice weighing 20–24 g from the same genetic background were used. They were kept in a room maintained at 21 ± 2°C with free access to a standard laboratory diet and tap water. All procedures were approved by the Nagasaki University Animal Care Committee and complied with the recommendations of the International Association for the Study of Pain [12]. Partial ligation of the sciatic nerve of the mice was performed under pentobarbital (50 mg/kg i.p.) anesthesia, following the methods of Malmberg and Basbaum [13]. In thermal paw withdrawal tests, nociception was measured as the latency to paw withdrawal evoked by exposure to a thermal stimulus [5,14]. Unanesthetized animals were placed in plexiglas cages on top of a glass sheet and an adaptation period of 1 hour was allowed. A thermal stimulator (IITC Inc., Woodland Hills, CA, USA) was then positioned under the glass sheet and the focus of the projection bulb was aimed exactly at the middle of the plantar surface of a particular paw. Paw pressure tests were performed as described previously [5,15]. Mice were placed into a plexiglas chamber on a 6 × 6-mm wire mesh grid floor and allowed to acclimatize for 1 hour. A mechanical stimulus was then delivered onto the middle of the plantar surface of the right hindpaw using a Transducer Indicator (Model 1601; IITC Inc., Woodland Hills, CA, USA). Electrodes (Neurotron Inc., Baltimore, MD) were attached to the right plantar surface and instep of a particular paw, as previously described [16]. Transcutaneous nerve stimuli with two sine-wave pulses (250 and 2000 Hz) were applied using a Neurometer CPT/C (Neurotron Inc. Blatimore, MD, USA). The minimum intensity (μA) at which each mouse withdrew its paw was defined as the current stimulus threshold. Stimuli were applied at 10-minute intervals. Investigators blinded to the phenotype of a gene carried out all experiments. Statistical analyses were performed using Student's *t*-test. Significance was set at p < 0.05.

**Figure 1:**
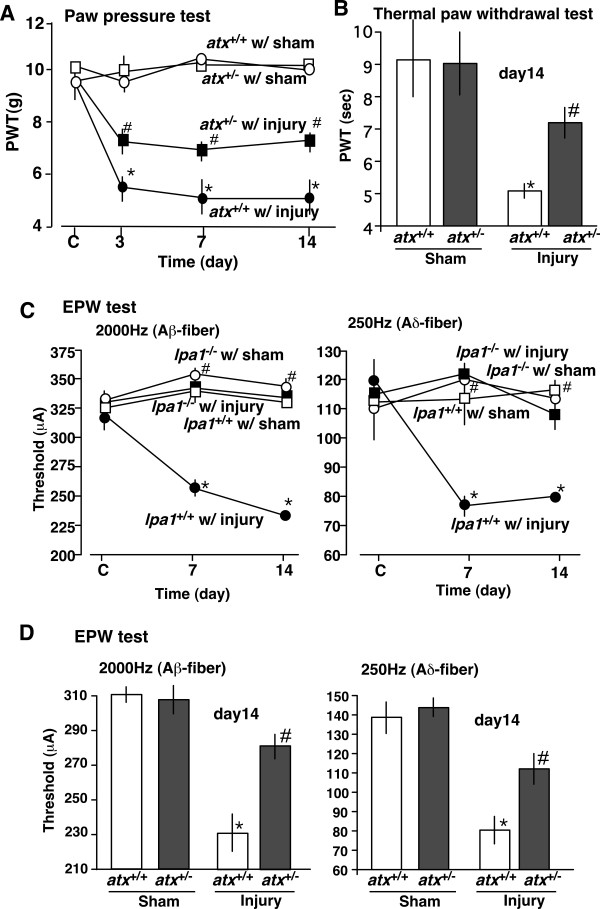
**Partial blockade of neuropathic pain in *atx*^+/- ^mice**. (A, B) Partial blockade of mechanical allodynia and hyperalgesia in *atx*^+/- ^mice. Paw pressure tests (A) were carried out on days 3, 7 and 14 after the nerve injury, while thermal paw withdrawal tests (B) were carried out on day 14 after the nerve injury. (C, D) Complete and partial blockade of Aβ (2000 Hz)- and Aδ (250 Hz)-fiber stimulation-induced hypersensitivities in *lpa*_*1*_^-/- ^(C) and *atx*^+/- ^(D) mice, respectively. Behavior experiments were carried out on days 7 and 14 (C) and day 14 (D) after the nerve injury. All data represent the mean ± SEM from 5–6 separate experiments. *p < 0.05 compared with sham-operated mice; #p < 0.05 compared with wild-type mice with nerve injury.

In paw pressure tests, partial sciatic nerve injury in wild-type (*atx*^+/+^) mice caused robust mechanical allodynia starting from day 3 after the nerve injury and persisting until at least day 14 (Figure 1A), consistent with a previous report [5]. There was no significant difference in the basal thresholds between heterozygous (*atx*^+/-^) and wild-type (*atx*^+/+^) mice. As shown in Figure 1A, the degree of mechanical allodynia was less evident in *atx*^+/- ^mice than in *atx*^+/+ ^mice. The threshold in *atx*^+/- ^mice with injury was between the level in sham-operated *atx*^+/+ ^mice or *atx*^+/- ^mice and that in *atx*^+/+ ^mice with injury, and the differences from these other groups were statistically significant for at least 14 days. Similar results were observed when nerve injury-induced thermal hyperalgesia was evaluated (Figure 1B).

We previously developed a nociceptive test (EPW test) to evaluate nociceptive paw-withdrawal behavior in response to electrical stimuli with different frequencies in a Neurometer^®^, an apparatus that selectively activates sensory neurons by using sine-wave pulses of different frequencies [16]. As shown in Figure 1C (*left panel*), the threshold for paw withdrawal upon 2000-Hz electrical stimulation, which is supposed to stimulate Aβ fibers, was approximately 320 μA in wild-type *lpa*_*1 *_^+/+ ^mice. Partial sciatic nerve injury in wild-type (*lpa*_*1*_^+/+^) mice caused a significant decrease in the withdrawal threshold to 2000 μA at day 7, which persisted until day14 (Figure 1C, *left panel*). Although there was no significant difference in the basal thresholds between knockout mice (*lpa*_*1*_^-/- ^mice) and wild-type mice, the hypersensitivity was completely abolished at days 7 and 14 in *lpa*_*1*_^-/- ^mice. On the other hand, the hypersensitivity to 2000-Hz electrical stimulation at day 14 after the nerve injury was partially, but significantly, attenuated in *atx*^+/- ^mice, which showed no significant change in the threshold without injury (Figure 1D, *left panel*). Quite similar results were observed when the withdrawal responses induced by 250-Hz electrical stimulation, which is supposed to stimulate Aδ fibers, were evaluated in *lpa*_*1*_^-/- ^mice and atx^+/- ^mice with and without nerve injury (Figure 1C and 1D, *right panels*).

In the present study, we used *atx*^+/- ^heterozygous mutant mice, since *atx*^-/- ^homozygous mutant mice were reported to die at the early stage of embryogenesis [10]. Therefore, these heterozygous mice are expected to have half the level of ATX expression, and indeed they were reported to show 50% lysoPLD activity relative to wild-type mice [10]. This finding is consistent with the present study, in which *atx*^+/- ^heterozygous mice showed partial attenuation of nerve injury-induced neuropathic pain, as observed for conventional mechanical allodynia and thermal hyperalgesia (Figure 1A), which are mediated through LPA_1 _receptor activation [5].

On the other hand, nerve injury is known to cause functional changes in myelinated A-fibers, such as demyelination, and upregulation of Ca^2+^channel α2δ-1 subunits and sodium channels in medium/large neurons of the dorsal root ganglion would underlie the molecular mechanisms for neuropathic pain. Previously, we demonstrated that nerve injury causes hypersensitization of myelinated Aβ- and Aδ-fiber functions in an electrical stimuli-induced paw flexion (EPF) test, which is a modified EPW test [17]. Here, we found that hypersensitization of myelinated Aβ- and Aδ-fiber functions following nerve injury was observed in the EPW test, and mediated through LPA_1 _receptor activation (Figure 1C). Consequently, we carried out further tests to clarify the involvement of ATX in Aβ- and Aδ-fiber hypersensitization. The *atx*^+/- ^heterozygous mice showed significant and partial attenuation of these phenomena. It is well known that LPA is mainly produced via two major pathways, namely LPC conversion mediated by activation of ATX and phosphatidic acid conversion mediated by activation of phospholipase A_2 _(PLA_2_) [6,7]. However, all the findings in the present report suggest that nerve injury-induced LPA production mainly occurs through LPC conversion mediated by activation of ATX.

There are reports that LPC is produced under physiological and pathological conditions [18,19]. Furthermore, LPC treatment of the saphenous or sciatic nerve induced neuropathic pain-like behaviors, such as mechanical allodynia and thermal hyperalgesia, as well as demyelination and upregulation of pain-related proteins in the dorsal root ganglion [20]. More recently, we found that i.t. injection of LPC induces neuropathic pain-like behaviors through ATX-LPA_1 _receptor signaling, since these behaviors were completely abolished in *lpa*_*1*_^-/-^mice and partially blocked in *atx*^+/- ^heterozygous mutant mice [21]. Therefore, LPC is involved in neuropathic pain.

We previously reported that i.t. injection of an antisense oligonucleotide for LPA_1 _receptor or inhibitors of RhoA/ROCK, one of the downstream signaling molecules of LPA_1 _receptor, completely abolished nerve injury-induced neuropathic pain [5]. Furthermore, i.t. injection of LPA mimics nerve injury-induced neuropathic pain. Therefore, nerve injury seems to cause LPA production in the spinal cord. ATX protein is present in the cerebrospinal fluid (CSF) and has lysoPLD activity to convert LPC into LPA [22]. On the other hand, LPC is not present in CSF [22]. Therefore, nerve injury would produce LPC in the spinal cord, which would subsequently be hydrolyzed by ATX to form LPA. Experiments to evaluate LPA and LPC production following nerve injury and clarify the relationship of ATX to LPA production are the next important issues to be addressed.

In addition to the lysoPLD activity to convert LPC to LPA, ATX also possesses activity to convert sphingosylphosphorylcholine to bioactive sphingosine-1-phosphate [23]. However, we previously reported that i.t. injection of sphingosine-1-phosphate did not cause neuropathic pain-like allodynia or hyperalgesia [5]. These findings suggest that the marked reduction of neuropathic pain in *atx*^+/- ^mice can be attributed to a reduction in LPA production following nerve injury.

In summary, we have demonstrated that LPA biosynthesis by ATX is the source of LPA for LPA_1 _receptor-mediated neuropathic pain. Therefore, targeted inhibition of ATX-mediated LPA biosynthesis as well as LPA_1 _receptor and its downstream pathways may represent a novel way to prevent nerve injury-induced neuropathic pain.
